# Sofosbuvir (SOF) Suppresses Ledipasvir (LDV)-resistant Mutants during SOF/LDV Combination Therapy against Genotype 1b Hepatitis C Virus (HCV)

**DOI:** 10.1038/s41598-017-15007-2

**Published:** 2017-10-31

**Authors:** Ashley N. Brown, Lin Liu, Jaime L. Rodriquez, Lisa Zhao, Layla Schuster, Eric Li, Gary P. Wang, Michael N. Neely, Walter Yamada, George L. Drusano

**Affiliations:** 1 Institute For Therapeutic Innovation, Department of Medicine, University of Florida, 6550 Sanger Road, Orlando, FL 32827 United States; 2 Infectious Diseases and Global Medicine, University of Florida, Gainesville, FL United States; 3 Medical Service, North Florida/South Georgia Veterans Health System, Gainesville, FL United States; 4 Laboratory of Applied Pharmacokinetics and Bioinformatics (LAPKB), Children’s Hospital Los Angeles Saban Research Institute, Los Angeles, CA United States

## Abstract

Our objective was to identify drug interactions between ledipasvir (LDV) and sofosbuvir (SOF) against a genotype 1b replicon to determine optimal exposures for each agent that will maximize antiviral activity against susceptible and drug-resistant subpopulations. LDV and SOF were evaluated using a fully factorial experimental design in the BelloCell system. Replicon levels and drug-resistant variants were quantified at various times post-therapy for 14 days and a high-dimensional mathematical model was fit to the data. Mutations associated with SOF resistance were not detected; but LDV-resistant mutants were selected and mutant subpopulations increased as exposure intensity increased. Combination therapy was additive for the total replicon population and the LDV-resistant population, but a threshold concentration of 100 ng/ml of SOF must be attained to suppress LDV-resistant subpopulations. These novel findings hold important implications for not only improving therapeutic outcomes, but also maximizing the clinical utility of LDV and SOF combination regimens.

## Introduction

Hepatitis C virus (HCV) infection is a significant global public health problem that affects nearly 3% of the world’s population. Approximately 70% of infected individuals will develop chronic HCV infections, which can lead to cirrhosis, liver cancer, liver failure, and eventually death. Chronic HCV is one of the major causes of hepatocellular carcinoma in the world and is the leading reason for liver transplantations in many countries, including the United States^1,2^. Liver-related morbidities and mortalities associated with infection can be prevented with successful antiviral therapy^3–5^. Thus, antiviral therapy plays a crucial role in the management of HCV.

The development and clinical implementation of direct-acting antivirals (DAAs) has revolutionized the treatment landscape for HCV. DAAs offer several advantages over traditional interferon-based regimens including oral availability, shorter durations of therapy, as well as significantly improved efficacy, tolerability, and safety profiles^6–11^. Despite these therapeutic improvements, drug resistance continues to be a major obstacle to the success of DAA regimens. Due to robust HCV replication kinetics and the error-prone nature of the RNA-dependent RNA polymerase, DAA-resistant viruses have been shown to emerge in patients under the selection pressure of antiviral therapy^12–16^. Combination therapy with two or more DAAs from different drug classes is an established approach for preventing the emergence of resistance in HCV-infected patients^17–21^. However, in order for combination therapy to be maximally effective, it is imperative to choose the appropriate combination of DAAs that will maximize/accelerate viral eradication as well as prevent resistance emergence.

Ledipasvir (LDV; NS5A inhibitor) and sofosbuvir (SOF; nucleotide NS5B inhibitor) are two DAAs that are approved in combination in the United States and Europe for the treatment of chronic HCV. This combination (Harvoni^®^) has had great clinical success against genotype 1 infections, with rates of sustained virologic response (SVR) greater than 90% in both clinical trials and real-world effectiveness analyses^6,22,23^. Treatment failures are often associated with the emergence of resistance-associated variants (RAVs) or resistance-associated substitutions (RAS)^24–28^. Consequently, it is important to understand exposure-response relationships for viral inhibition and resistance suppression for LDV and SOF in order to sustain or even improve upon therapeutic outcomes.

In this investigation we examined LDV and SOF, alone and in combination, against a genotype 1b HCV replicon cell line to define drug-drug interactions for antiviral effect and resistance suppression. These results will allow for the identification of optimal exposures for both LDV and SOF that will maximize antiviral activity and minimize the emergence of RAVs or RAS. This experimental approach can be utilized to select and rationally design optimal combination regimens for the treatment of HCV that will maximize therapeutic benefits in patients.

## Results

### Effectiveness of SOF and LDV against a genotype 1b HCV replicon in a plate screening assay

The EC_50_ value for SOF against the genotype 1b HCV replicon-bearing 2209-23 cell line was 45.52 ng/ml (95% C.I.: 37.10–50.69 ng/ml) and the EC_50_ value for LDV was 1.421 pg/ml (95% C.I.: 0.979–1.862 pg/ml) after three days of treatment in a 96-well plate.

### Effectiveness of SOF and LDV as combination therapy against a genotype 1b HCV replicon in the BelloCell system

We evaluated the activity of SOF and LDV as mono- and combination therapy against 2209-23 cells in the BelloCell system^29^ to define the drug-drug interactions for antiviral effect. For these studies, we implemented a fully factorial experimental design in which three exposures of SOF and LDV were assessed as monotherapy in addition to every possible combination of exposures (Table 1). The SOF and LDV exposures utilized in these experiments were selected from the plate screening assay results described above, as the concentrations employed for SOF (22.64 ng/ml, 45.52 ng/ml, and 295.9 ng/ml) and LDV (0.67 pg/ml, 1.42 pg/ml, and 9.97 pg/ml) correspond to the EC_25_, EC_50_, and EC_95_ values, respectively, determined from these assays.

**Table 1:** SOF and/or LDV regimens evaluated in the BelloCell System against a genotype 1b HCV replicon cell line.

	[Sofosbuvir] (ng/ml)^a^			
[Ledipasvir] (pg/ml)^a^	0	EC_25-SOF_	EC_50-SOF_	EC_95-SOF_
	EC_25-LDV_	EC_25-LDV_EC_25-SOF_	EC_25-LDV_EC_50-SOF_	EC_25-LDV_EC_95-SOF_
	EC_50-LDV_	EC_50-LDV_EC_25-SOF_	EC_50-LDV_EC_50-SOF_	EC_50-LDV_EC_95-SOF_
	EC_95-LDV_	EC_95-LDV_EC_25-SOF_	EC_95-LDV_EC_50-SOF_	EC_95-LDV_EC_95-SOF_

^a^Concentrations were determined using a 96-well plate drug screening assay.

**Figure 1:**
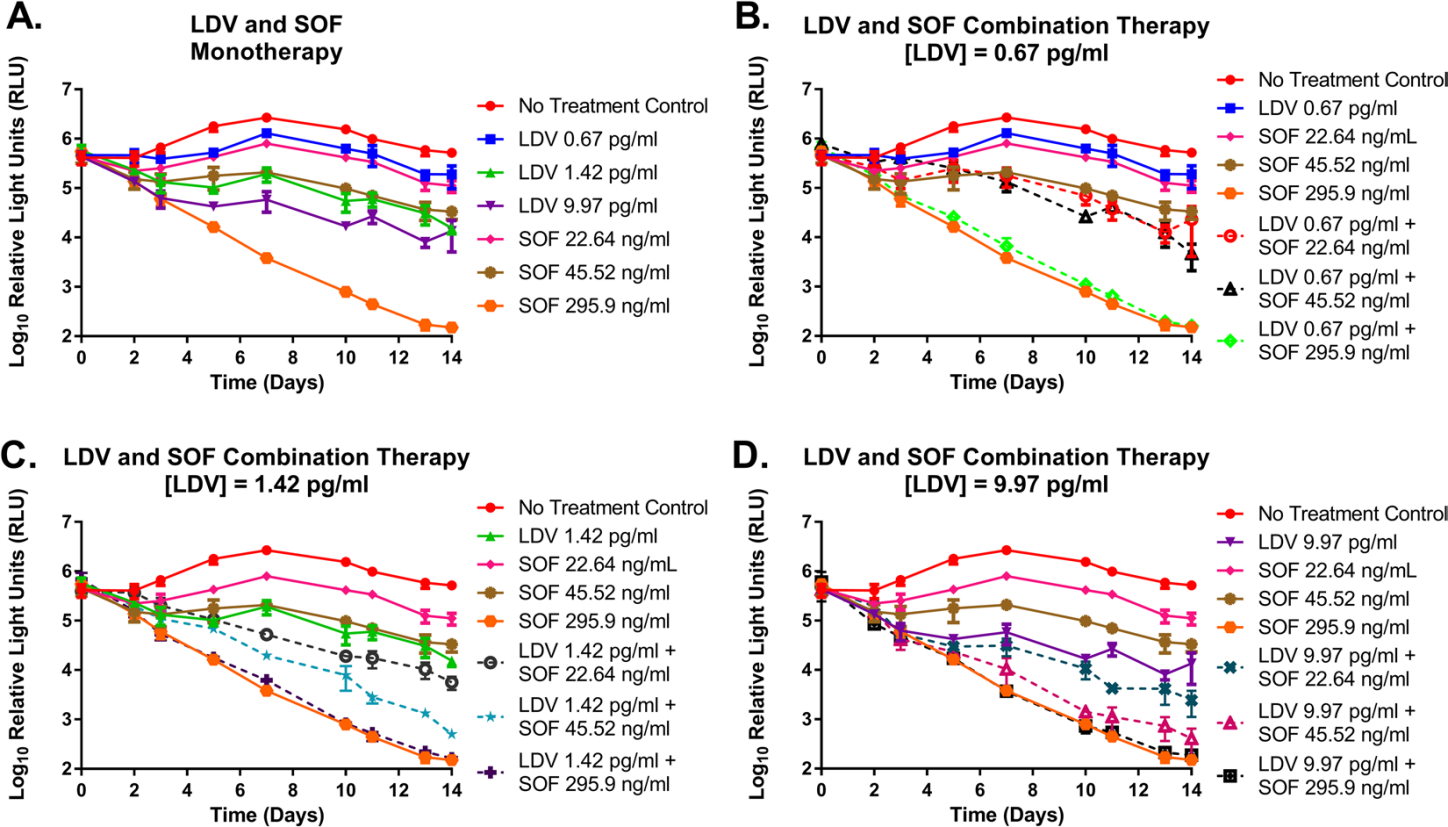
Antiviral activity of LDV and SOF as monotherapy (**A**) and combination therapy (**B**–**D**) against a genotype 1b HCV replicon cell line in the BelloCell system. Replicon levels are reported as Log_10_ Relative Light Units (RLU) as determined using a *Renilla* luciferase assay. Each data point represents the geometric mean of three independent samples and error bars correspond to one standard deviation.

As monotherapy, SOF and LDV suppressed the genotype 1b replicon in a dose dependent manner and the degree of suppression was similar for the two drugs in the EC_25_ and EC_50_ treatment arms (Fig. 1A). Maximal antiviral effect was achieved with the EC_95_ concentration of SOF and suppressed luciferase activity by approximately 4-log_10_ at day 14 relative to the no-treatment control. The EC_95_ regimen for LDV, on the other hand, was only marginally more effective than the EC_50_ concentration (Fig. 1A). Antiviral activity was enhanced when SOF and LDV were administered as combination therapy relative to the monotherapy arms and effectiveness increased when higher concentrations of SOF and LDV were used in combination (Fig. 1B–D). However, combination regimens containing 295.9 ng/ml (EC_95_) of SOF performed as well as the corresponding SOF monotherapy arm due to the fact that maximal antiviral activity was attained at this SOF concentration.

**Figure 2:**
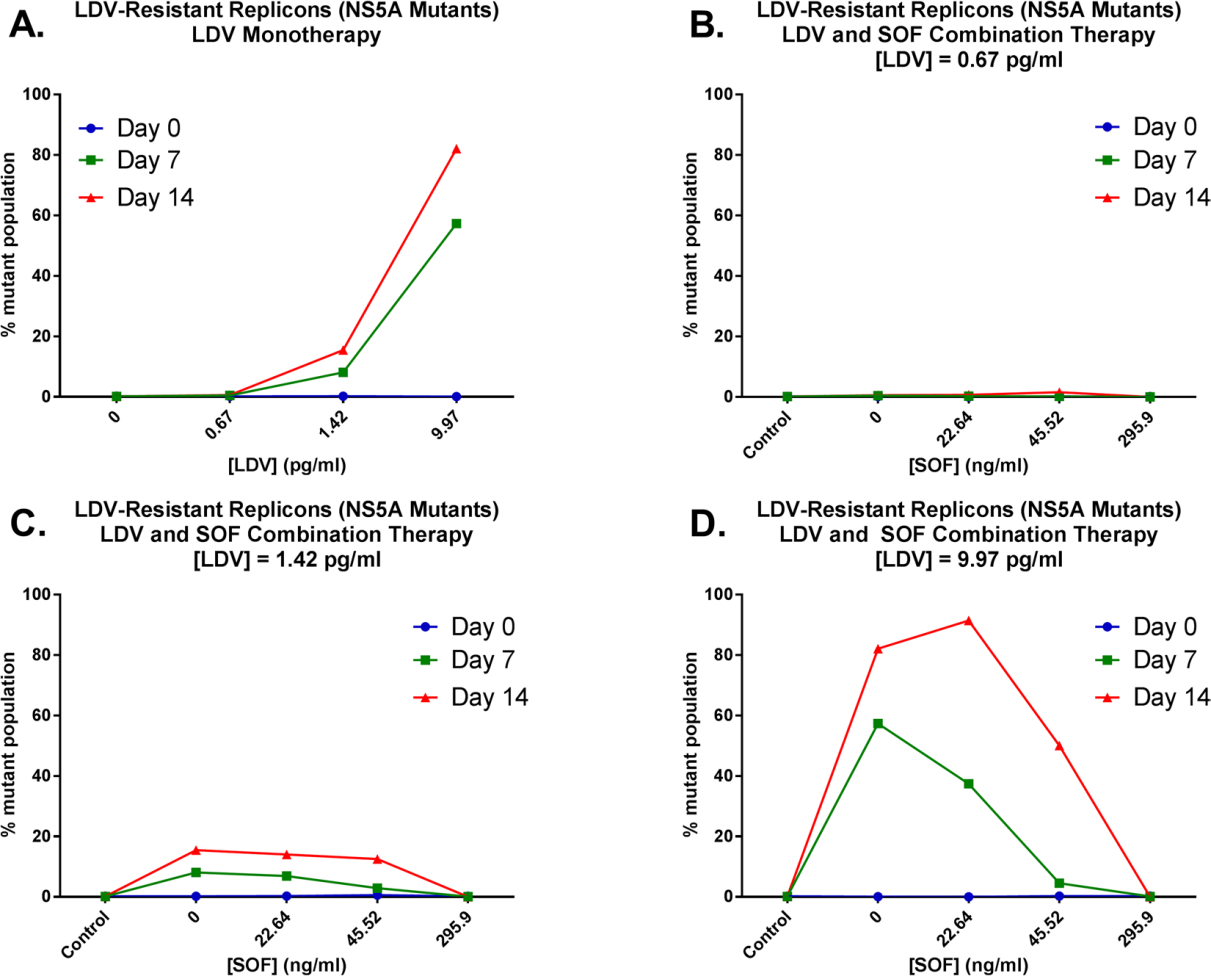
The percentage of genotype 1b HCV replicon subpopulations that harbor RAVs to LDV at amino acid positions 31 and/or 93 at baseline (Day 0; blue line), day 7 (green line), and day 14 (red line) post-LDV exposure. Mutant genotypes were identified using single variant sequencing methods.

**Figure 3:**
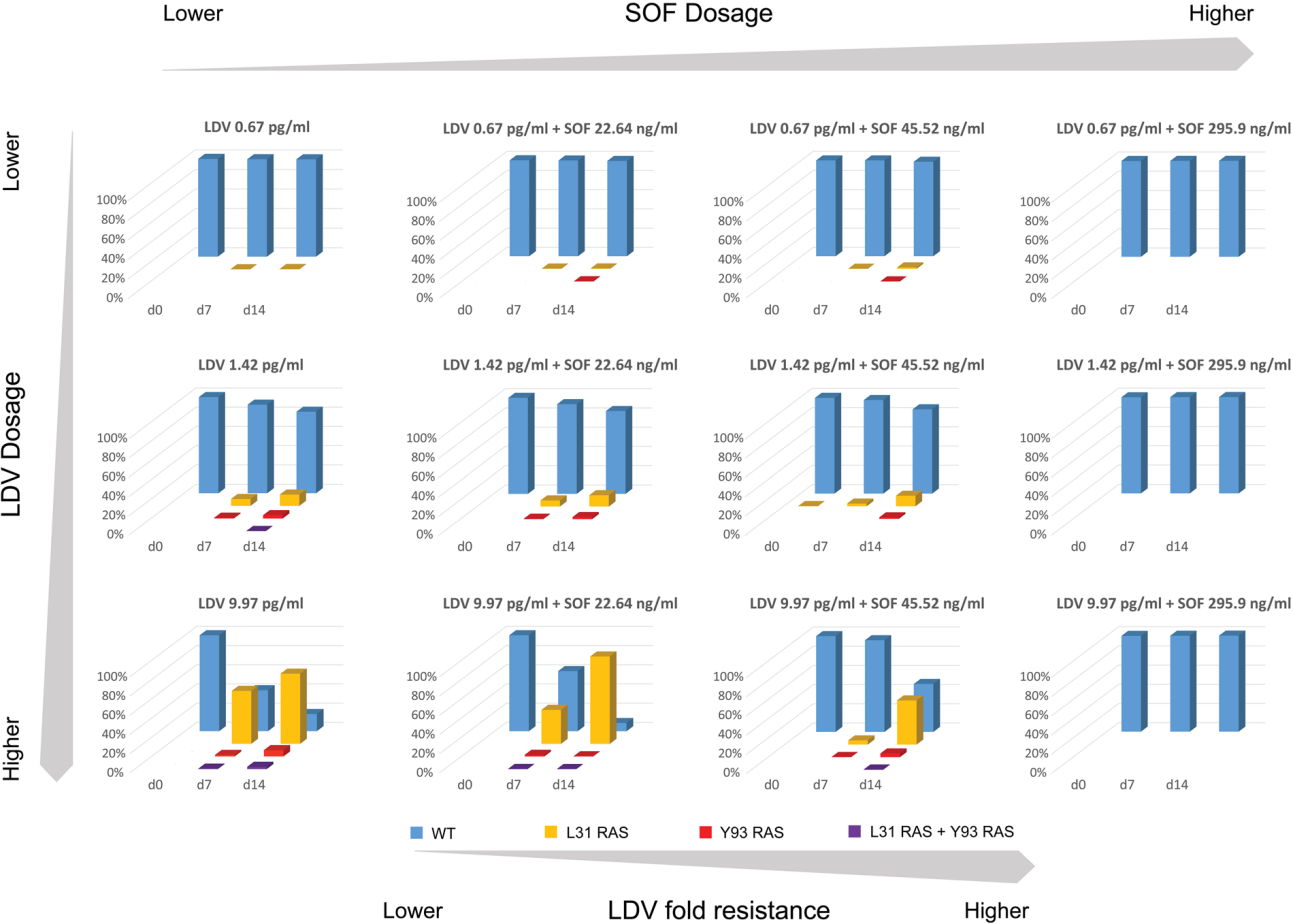
Abundance and linkage of LDV-associated RAVs identified at days 0, 7, and 14 post-LDV therapy with or without various concentrations of SOF in the BelloCell system. NS5A positions L28, P29, R30, L31, P32, P58, E62, A92, and Y93 based on HCV CON-1^45^ were queried and RAVs were identified using single-variant sequencing^43^ as described in the methods section. Blue bars correspond to wild-type (WT) replicons, yellow bars represent replicons with a RAV at the L31 amino acid position but are WT at the Y93 position, orange bars denotes replicons with a RAV at the Y93 position but are WT at the L31 position, and red bars are double-mutant replicons that have RAVs at both the L31 and Y93 positions.

In addition to replicon kinetics, the selection of NS5A and NS5B RAVs under the drug pressure of each experimental regimen was evaluated using single-variant sequencing at days 0, 7, and 14 post-treatment (Fig. 2). Resistance profiles for SOF and LDV are independent and cross-resistance between these two agents has not been reported^30^. Thus, SOF is effective against LDV-specific RAVs and vice versa. No RAVs that are associated with resistance to SOF were detected at NS5B RAVs sites (amino acid L159 and S282 based on HCV CON-1) in any regimen throughout the entire duration of the study. RAVs associated with LDV resistance (NS5A L31 and/or Y93 substitutions) were readily identified in replicons harvested from experimental arms treated with LDV (Figs 2A–D and 3). LDV monotherapy greatly influenced the proportion of mutant subpopulations, as the percentage of replicon mutants increased with increasing LDV concentrations (Fig. 2A). The highest concentration of LDV evaluated in this study (EC_95_ = 9.97 pg/ml) resulted in peak mutant subpopulations equivalent to 82.1% at day 14 compared to 15.5% mutants and 0.6% mutants in the 1.42 pg/ml (EC_50_) and 0.67 pg/ml (EC_25_) arms, respectively (Fig. 2A). LDV concentrations also affected the position, abundance and linkage of RAVs that were selected in the mutant subpopulation (Fig. 3). While replicons harboring mutations in NS5A amino acid position 31 were most prevalent in both the 1.42 pg/ml (EC_50_) and 9.97 pg/ml (EC_95_) experimental arms, L31 + Y93 double mutants were observed only in the 9.97 pg/ml (EC_95_) LDV treatment arm (Fig. 3).

SOF was effective in suppressing LDV-associated mutant replicons when used in combination with LDV, but only when sufficient concentrations of SOF were used (Figs 2C,D and 3). This was best demonstrated in experimental arms that received 9.97 pg/ml (EC_95_) of LDV, the concentration that selected for the highest proportion of mutant subpopulations (Fig. 2D). The addition of 22.64 ng/ml (EC_25_) SOF to 9.97 pg/ml LDV did not inhibit the replication of LDV-associated resistant replicons, as the percentage of mutants in this regimen were similar, and even slightly higher, to those that reported in the LDV monotherapy arm at day 14. SOF at 45.52 ng/ml (EC_50_) provided some control over LDV-resistant replicons compared to LDV monotherapy, reducing the mutant subpopulation to 50.1%. Replicons containing LDV-associated RAVs were completely suppressed in the presence of SOF at 295.9 ng/ml (EC_95_) (Figs 2C,D and 3).

### Mathematical modeling of SOF and LDV combination therapy

**Figure 4:**
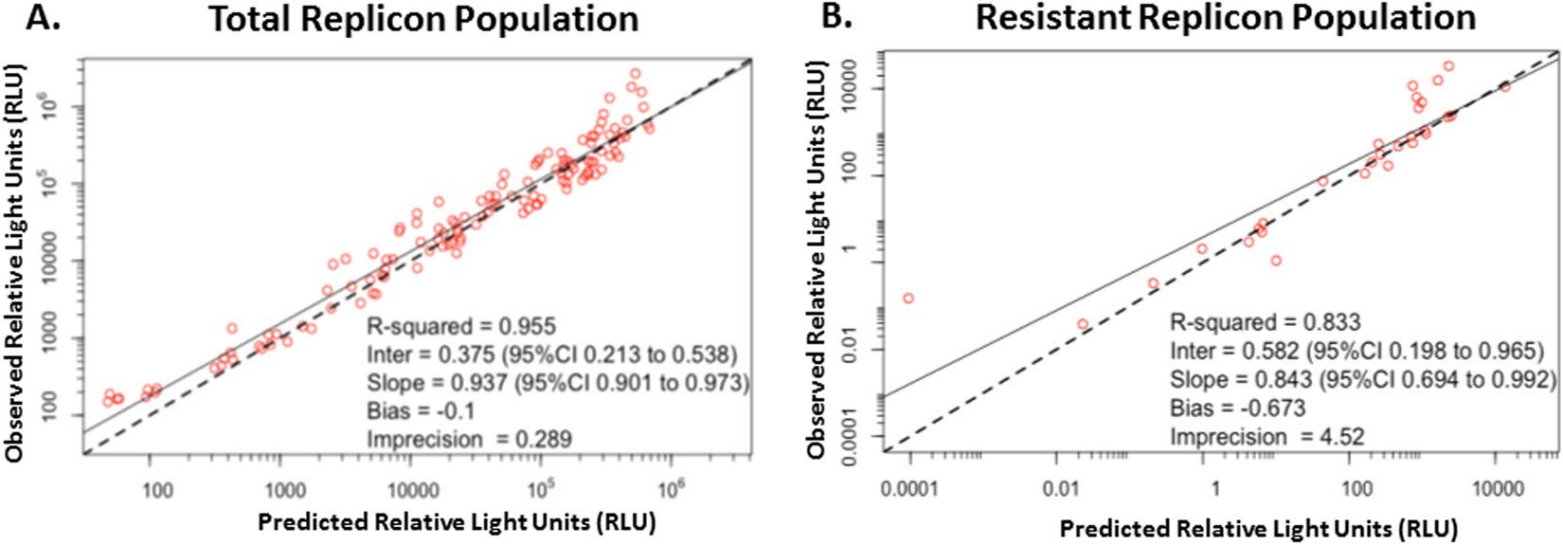
Predicted-versus-observed plots for genotype 1b HCV replicon levels, reported as relative light units (RLU), from the total population (**A**) and the LDV-resistant population (**B**) after treatment with SOF and/or LDV in the BelloCell system.

A high dimensional mathematical model (see methods) was simultaneously fit to the *Renilla* luciferase output (Log_10_ Relative Light Units (RLU)) from the total replicon population and the LDV-associated resistant replicon subpopulation to describe exposure-response relationships for SOF + LDV as combination therapy against genotype 1b HCV replicons over time as well as identify drug-drug interactions between SOF and LDV for antiviral effect. The model fit the data well and resulted in precise and unbiased curve fits, as demonstrated by the predicted-versus-observed plots for the total replicon population and resistant replicon population (Fig. 4A,B). The Predicted Observed plot yielded an *r* ^2^ value of 0.955, a slope of 0.937, and a y-intercept of 0.375 for the total replicon population and an *r* ^2^ value of 0.833, a slope of 0.843, and a y-intercept of 0.582 for the LDV-associated resistant replicon population. Both regressions were highly statistically significant, with p values <<0.001.

The median parameter estimates for the combination therapy model are shown in Table 2. The model estimated that LDV-associated resistant replicons were 4.5-fold less susceptible to LDV compared to the susceptible replicon population due to the shift in EC_50_ value in the resistant population (EC_50-D1r_ = 18.625 pg/ml versus EC_50-D1s_ = 4.147 pg/ml). Additionally, RAVs to LDV did not compromise replicon biofitness, as the estimate for K_turn-D1r_ was higher than that reported for K_turn-s_. The drug interaction parameter for antiviral effect against the susceptible replicon population is positive with a 95% confidence interval that crosses 0 (α_s_ = 0.315, 95% C.I.: −0.15–2.4) indicating that additivity is achieved when SOF and LDV are used in combination. SOF and LDV are also additive as combination therapy against LDV-associated resistant replicons (α_D1r_ = 0.334; 95% C.I. = −2.6–0.63).

**Table 2:** Population median parameter estimates for the combination therapy mathematical model.

Parameter	Symbol (unit)	Population median estimates	
		Median	95% C.I.^a^
Maximal amount of replicon levels	POPMAX (Log_10_ RLU)	21.446	
First-order replicon turnover rate for susceptible replicon population	K_turn-s_ (day^−1^)	0.650	
First-order rate of loss of replicon from cells for susceptible replicon population	K_loss-s_ (day^−1^)	0.670	
Effective concentration 50 value of LDV for susceptible replicon population	EC_50-D1s_ (pg/ml)	4.147	
Effective concentration 50 value of SOF for susceptible replicon population	EC_50-D2s_ (pg/ml)	57,356	
Drug interaction term for effect for susceptible replicon population	α_s_	0.315	−0.15–2.4
First-order replicon turnover rate for replicons resistant to LDV^b^	K_turn-R1_ (day^−1^)	0.815	
First-order rate of loss of replicon from cells for LDV-resistant replicons	K_loss-R1_ (day^−1^)	0.793	
Effective concentration 50 value of LDV for replicons resistant to LDV	EC_50-D1R_ (pg/ml)	18.625	
Drug interaction term for effect for LDV-resistant replicons	α_D1r_	0.334	−2.6–0.63
Hill constant for susceptible replicon population to LDV	H_D1-s_	0.997	
Hill constant for suscpetible replicon population to SOF	H_D2-s_	2.985	
Hill constant for replicons resistant to LDV	H_D1-R_	3.253	

^a^95% confidence Interval (C.I.) was determined by bootstrap analysis. ^b^Replicons harboring RAVs to SOF were not detected in this study.

**Figure 5:**
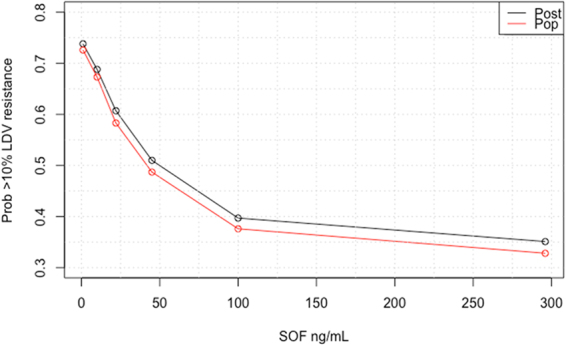
Simulation plot predicting the probability of LDV-resistant mutant replicon amplification with different SOF concentrations during SOF + LDV combination therapy. For the simulations, a baseline replicon level of 500,000 RLU was used with 0.2% of the population representing replicons harboring RAVs to LDV. The red line corresponds to the simulation run prior to Bayesian estimation and the black line represents Bayesian posterior simulation.

We conducted simulations using the median parameter estimates to predict the concentration of SOF that decreases the probability of amplifying LDV-associated resistant replicons. The simulations showed that the probability of LDV-resistance substantially increases when SOF concentrations fall below 100 ng/ml (Fig. 5). These results suggest that a threshold of 100 ng/ml of SOF must be achieved to in order to suppress LDV-associated resistant variants.

## Discussion

The presence of RAVs, either pre-existing or resulting from treatment, can negatively impact therapeutic outcomes in patients infected with HCV^24–28,31^. Thus, the long-term clinical success of antiviral therapy hinges on the ability of the regimen to rapidly suppress HCV and prevent the emergence of drug-resistant variants. Combination therapy with two or more DAAs is one strategy for achieving sustained virologic response and there are currently several combination regimens available for the treatment of chronic HCV. However, previous clinical studies have shown that all combinations are not created equal and that administering multiple DAAs in combination does not guarantee resistance suppression^32–34^. Here we describe an experimental approach that allows for the evaluation of DAAs in combination to inhibit HCV replication and suppress resistance emergence. Furthermore, we describe a high dimensional mathematical model that provides valuable information regarding drug-drug interactions for antiviral effect and exposure-response relationships for the agents in combination. This methodology can be used to rationally identify optimal combinations of DAAs and predict the exposures of each agent required to maximize effectiveness.

The combination of LDV and SOF is arguably one of the most successful DAA regimens on the market today. We evaluated LDV and SOF, either alone or in combination, in the BelloCell system against a genotype 1b HCV replicon to identify the antiviral properties that make this regimen so clinically effective. As monotherapy, maximal antiviral activity was achieved for most LDV and SOF regimens early, with replicons levels plateauing at day 3 post-therapy and remaining relatively stable throughout the 14-day experiment. This is in contrast to the EC_95_ SOF experimental arm in which replicon levels continued to decrease over time and maximal antiviral activity was attained with this regimen (Fig. 1A). Sequencing analyses revealed that differences in antiviral kinetics between LDV and SOF, particularly at the EC_95_ concentrations, may be attributed to the emergence of resistance. LDV has a low genetic barrier to resistance; consequently, several LDV-associated RAVs have been described in patients with genotype 1 infections^16,25,28^. Moreover, these LDV RAVs are biofit and have been shown to persist in patients for years^35^. SOF, on the other hand, exhibits a high genetic barrier to resistance and RAVs to SOF are rarely detected in HCV-infected individuals, even after treatment failure^10,36–38^. Our findings mirror these clinical observations in regards to resistance. RAVs to SOF were not detected in our study, but LDV-associated resistant variants were rapidly amplified under the selection pressure of LDV and resistant subpopulations were more prevalent with exposures of LDV at higher concentrations (Fig. 2). This “inverted-U”-like response, which our group has described previously for other antiviral and antibacterial agents^29,39,40^, is defined by the presence of a low proportion of drug-resistant mutants at lower concentrations of drug. This likely occurs because the decreased drug pressure allows for wild-type replicons to replicate. As drug pressure increases, the wild-type population is suppressed but the mutant subpopulations prevail. However, LDV concentrations in our evaluation were not high enough to control mutant subpopulations which were overall 4.5-fold less susceptible to LDV than wild-type replicons, as demonstrated by the mathematical model.

It is also of interest that the concentrations of LDV influenced the genotypes and the abundance of mutants that were selected. The mutant variants identified in our study (L31 and Y93) are also the primary RAVs described in patients with genotype 1b infections treated with LDV^16^. However, different RAVs confer varying degrees of resistance to LDV. For example, substitutions at L31 were the most prevalent in our study across all LDV exposures and are associated with a 2.5- to 100-fold decrease in susceptibility to LDV^25^. Mutants at the Y93 position were more prevalent, albeit at low frequencies, with higher concentrations of LDV and have been shown previously to confer more than 1000-fold LDV resistance^25^. Finally, L31 + Y93 double mutants were detected only at the highest exposure of LDV evaluated and presumably confer even higher levels of resistance^41^. As the concentration of SOF increases from EC_25_ to EC_95_, the abundance of both single (L31, Y93) and double (L31 + Y93) mutants decreases and RAVs were suppressed completely at SOF concentration of 295.9 ng/mL (EC_95_) (Fig. 3). RAV genotype has been shown to play an important role in therapeutic outcomes, as patients with LDV-associated RAVs that conferred more than 100-fold resistance had lower rates of SVR with LDV and SOF combination therapy in clinical trials^25^. The mathematical estimate of LDV-resistance in this study corresponds to the overall population of mutants which is largely composed of the least-resistant L31 mutant; however, RAVs that confer substantially higher levels of resistance are also present, but at lower proportions. Therefore, it is important to understand the influence of LDV exposure on the prevalence and type of RAVs that emerge under selection pressure associated with therapy and how this influence may affect therapeutic outcomes.

Combination therapy with LDV and SOF in our experiments resulted in enhanced antiviral activity, interacting in an additive fashion for inhibition of the susceptible replicon population. LDV and SOF interactions were also additive against LDV-resistant subpopulations. However, when suboptimal levels of SOF were administered, LDV-resistant mutant subpopulations prevailed. Our results clearly demonstrate that a sufficient concentration of SOF must be present in order to suppress LDV-resistant replicons. Therefore, it must be emphasized that it is not sufficient to just have LDV and SOF in combination, but that each drug must be present at the appropriate exposure in order to optimally inhibit replicon replication and suppress resistance emergence.

We employed a mathematical model to conduct simulations in an attempt to identify a threshold value of SOF required to suppress LDV-resistance. Our findings illustrate that the probability of LDV-resistant mutants significantly increases when SOF concentrations fall below 100 ng/ml. This is a completely novel finding that may hold very important clinical implications, as treatment failures in patients treated with LDV/SOF combination regimen may be directly related to suboptimal SOF exposures in these individuals. When a fixed dose of a drug, like SOF, is administered to a population of patients, a range of exposures will be achieved in the population due to inter-patient pharmacokinetic variability. This means that a fraction of SOF exposures in a distribution of patients will not be high enough to prevent the replication of LDV-resistant viruses, ultimately resulting in failure. However, the true clinical translation of this 100 ng/ml concentration is difficult to interpret due to the mechanism of action of SOF. SOF is a nucleotide prodrug that must be metabolized into its active tri-phosphorylated form before it can exert antiviral activity. However, the intracellular kinetics of tri-phosphorylated SOF have not been well defined in humans. We are currently conducting studies and developing models to correlate concentrations of the parent SOF compound to intracellular concentrations of the active tri-phosphate metabolite. These studies will provide greater insight into the threshold of SOF that must be attained to prevent LDV-resistance. Until then, it would be worthwhile to examine SOF/GS-331007 (the major metabolite of SOF) concentration-time profiles in patients that exhibit treatment failures during LDV and SOF combination therapy in addition to viral resistance genotype.

For antiviral therapy to be successful for the treatment of HCV, the optimal agents at the appropriate concentrations must be included in the combination regimen. Despite the clinical success of LDV and SOF, treatment failures still occur and are most often associated with resistance. Here we describe a threshold of SOF that, if attained, can likely minimize treatment-related failures and ensure long-term utility of this regimen. The experimental and analytical approach employed in these studies can be applied to evaluate new combinations of DAAs and identify regimens that will maximize viral suppression, minimize resistance emergence, and potentially further shorten durations of therapy.

## Methods

### Cells and reagents

The 2209-23 cell line, which stably expresses a genotype 1b HCV replicon, has been described previously^42^. 2209-23 cells were maintained in high-glucose Dulbecco’s modified Eagle’s medium (DMEM; HyClone, Logan, UT) containing 4.0 mM _L_- glutamine and sodium pyruvate with 10% fetal bovine serum (FBS; HyClone), 1% penicillin-streptomycin solution (Hyclone), and 500 µg/ml of geneticin (G418; Invitrogen, Carlsbad, CA) at 37 °C, 5% CO_2_. Cells were passaged twice weekly using trypsin 0.05% with EDTA (Hyclone) to sustain subconfluency.

SOF and LDV were provided by Gilead Sciences, Inc. (Foster City, CA) as a powder. Drug stocks of 10 mg/ml were made for each agent by reconstituting the powder in dimethyl sulfoxide (DMSO) followed by sterile filtration through a 0.2 micron filter. Stocks were aliquoted and stored at −80 °C prior to use.

### Antiviral drug screening assays

The antiviral effectiveness of SOF and LDV against a genotype 1b HCV replicon was determined using a 96-well plate screening assay, as previously described^29^. Briefly, 2209-23 cells were seeded into white opaque 96-well plates at a final concentration of 5,000 cells per well. Cells were allowed to adhere to the 96-well plate for 24 h at 37 °C, 5% CO_2_. Drugs were diluted to various concentrations in assay medium (2209-23 cell culture medium described above without G418) containing 1% DMSO (to ensure drug solubility) and 0.1 mls of each dilution, including a no-treatment control, was added to the 96-well plate in triplicate. Cells and drug were incubated for 72 h at 37 °C, 5% CO_2_. Luciferase activity was measured using the *Renilla* luciferase assay system (Promega; Madison, WI) and GloMax 96 Microplate Luminometer (Promega). EC_50_ values for SOF and LDV were calculated using GraphPad Prism software (GraphPad, La Jolla, CA).

### Antiviral Evaluations in the BelloCell System

The BelloCell system for anti-HCV evaluations is described elsewhere^29^. Briefly, 2209-23 cells were inoculated into 16 BelloCell-500P cell culture bottles (Chemglass Life Sciences; Vineland, NJ) at a concentration of 6 × 10^7^ cells per 500 mls of assay medium. Cells were allowed to attach to carrier flakes for 24 h at 37 °C, 5% CO_2_ prior to drug treatment. SOF and/or LDV were diluted in assay medium to the concentrations illustrated in Table 1 and administered into 15 BelloCell bottles via a continuous infusion using Masterflex L/S digital pumps (Cole-Parmer; Vernon Hills, IL). A final concentration of 1% DMSO was maintained in the assay medium to ensure compound solubility. One bottle served as a no-treatment control and was perfused with only assay medium supplemented with 1% DMSO. SOF and/or LDV-containing medium was made fresh every 48 h and added to the BelloCell system. Studies were conducted for 14 days. At various times post-drug exposure, carrier flakes were sampled from each BelloCell bottle and processed as previously described^29^. Replicon levels in all samples were measured simultaneously using the *Renilla* luciferase assay system.

### Single-Variant Sequencing (SVS) of NS5A and NS5B gene segments

Cellular RNA was extracted from 2209-23 cells harvested from the BelloCell system using an RNeasy minikit (Qiagen, Valencia, CA) following the manufacturers’ instructions and extracts were frozen at −80 °C until the end of the experiment. The HCV NS5A and NS5B gene segments were amplified and sequenced from extracted RNA using the SVS methods described previously^43^. Briefly, a random 12-nt sequence (i.e. tag) was incorporated into the 5′ end of the reverse-transcription (RT) primer to tag individual RNA templates during RT reaction, and this random sequence tag was flanked by a primer binding sequence on the 5′ end, which was required for the subsequent PCR reaction. Following RT, excess RT primers were removed using a Macherey-Nagel DNA purification column (Macherey-Nagel, Bethlehem, PA, USA). The cDNA was PCR amplified using barcoded primers, and tailed with Illumina adaptor sequences required for deep sequencing. PCR conditions were: initial denaturation at 94 °C for 2 min followed by 30 cycles of 94 °C for 20 s, 56 °C for 20 s, and 68 °C for 1 min, an extra 5 min at 68 °C was added at the end of amplification. All primers were designed based on HCV-CON1 (subtype 1b, NCBI Genbank accession number AJ238799)^44^. Final PCR products were purified using a gel extraction kit (Macherey-Nagel, Bethlehem, PA, USA), quantified using a Qubit kit (Life technologies), and pooled with equimolar concentrations. The concentration of the final DNA pool was quantified by real-time PCR using a SYBR Green qPCR kit (KAPA Biosystems). The DNA library was then prepared and sequenced on a benchtop MiSeq sequencer (Illumina, San Diego, CA) following manufacturer’s instructions. A Q30 filter was used to select for high quality reads, generating a total of 5.62 Gigabases of nucleotides. Insertions and deletions were found to be minimal in our sequence dataset.

### Bioinformatics analysis of HCV resistance variants

Illumina paired-end sequencing data were de-multiplexed into individual samples according to unique combinations of forward and reverse barcodes, followed by additional filtering criteria to select for high-quality reads. Each paired-end read was joined using FLASh (http://ccb.jhu.edu/software/FLASH/) with a minimum of 10 base overlap. Specifically, an average quality score of 30 for each paired end read was required, and primer sequences were used as quality control to exclude reads with mismatching sequences. For each sample, joined reads were grouped by unique 12-nt sequence tags, as each starting RNA template was labeled with a unique, random sequence tag that were introduced during RT. To determine the authentic sequence of each initial RNA template and correct for PCR amplification and sequencing errors, a consensus sequence was determined for each unique sequence tag based on an alignment of at least three reads using MAFFT (http://mafft.cbrc.jp/alignment/software/). Those with fewer than 3 reads per unique tag were discarded. Consensus sequences were then aligned against HCV CON-1 reference sequence using a 36 CPU Amazon Web Services EC2 Ubuntu Linux (version 14.04) instance (https://aws.amazon.com/ec2), and visualized using BioEdit (version 7.2.5.0; http://www.mbio.ncsu.edu/BioEdit/bioedit.html).

Translation of codons, calculation of resistance-associated substitutions (RASs), and linkage analysis of RASs were carried out using custom R scripts (https://www.r-project.org/) with the BioStrings package (http://bioconductor.org/packages/release/bioc/html/Biostrings.html). Amino acid substitutions at each position were identified by comparing translated consensus reads against HCV CON-1 reference, and the proportions of wild-type and RASs at each position were calculated. RASs associated with LDV and SOF resistance were defined according to Lontok *et al*.^45^, and were used for the resistance analysis. Linkage between RASs was determined by calculating the proportions of wild-type HCV CON-1 and variants carrying single, double or multiple RAS(s).

### Mathematical Modeling of Combination Therapy

We employed a population modeling approach to simultaneously identify drug-drug interactions for antiviral effect between SOF and LDV against multiple populations of replicons with varying susceptibilities to each drug. Because the concentrations of both drugs were held constant, and no subpopulations resistant to SOF were detected, the mathematical model consisted of only two simultaneous parallel inhomogeneous differential equations displayed below and was implemented using the Non-Parametric Adaptive Grid (NPAG) software program^46^.

1 $$\frac{d({N}_{s})}{dt}={N}_{s}x({k}_{turn-s}x{M}_{s}xG-{k}_{loss-s})$$

2 $$\frac{d({N}_{R1})}{dt}={N}_{R1}x({k}_{turn-R1}x{M}_{R1}xG-{k}_{loss-R1})$$

$G=(1\,-\,\frac{({N}_{S}+\,{N}_{R1})}{POPMAX})$; *M* = (1 − *E*) from the Greco URSA model

Differential equations 1 and 2 describe the replication and inhibition of the susceptible replicon population (equation 1) and replicon subpopulation that are resistant to LDV (equation 2). The nomenclature for the resistant replicon subpopulation is the same as for the total replicon population with the subscript changed from “s” (for susceptible) to “R1” (for resistant to LDV but susceptible to SOF). N_s_ represents the replicon levels from the susceptible population, k_turn-s_ is the first-order turnover rate constant for the susceptible replicon population, G is a logistic carrying function which allows the replicon population to achieve a stationary phase, k_loss-s_ represents the first-order rate of loss of the susceptible replicons from the cells, and M_s_ incorporates the Greco Universal Response Surface Approach (URSA) model, as previously described^47^, for the susceptible population. The equation for the Greco URSA model is shown below:

$$1=\frac{{D}_{1}}{E{C}_{50D1}{(\frac{E}{{E}_{con}-E})}^{1/{H}_{1}}}+\frac{{D}_{2}}{E{C}_{50D2}{(\frac{E}{{E}_{con}-E})}^{1/{H}_{2}}}+\frac{\alpha {D}_{1}{D}_{2}}{E{C}_{50D1}E{C}_{50D2}\,{(\frac{E}{{E}_{con}-E})}^{1{/2}_{{H}_{1}}+1{/2}_{{H}_{2}}}}$$

where D_1_ corresponds to the concentration of LDV and D_2_ represents the concentration of SOF. EC_50 D1_ is the concentration of LDV resulting in half maximal effect and EC_50 D2_ is the concentration of SOF resulting in half maximal effect. H_1_ and H_2_ represent the Hill’s constants for LDV and SOF, respectively and E_con_ refers to replicon levels in the absence of drug. Finally, α is the drug-drug interaction parameter for antiviral effect and defines whether synergy, additivity, or antagonism exists when LDV and SOF are used in combination. If the 95% confidence interval (CI) around α encompasses zero, the antiviral effect is considered to be additive. If α and its 95% CI do not cross zero and are positive, the effect is synergistic. If α and its 95% CI do not cross zero and are negative, the effect is antagonistic. This methodology allows us to explore combination chemotherapy for total replicon inhibition as well as suppression of drug-resistant replicon subpopulations.

### System outputs

The system outputs are Y(1) which is the Log_10_ RLUs associated with the susceptible replicon population and Y(2), which is the Log_10_ RLUs associated with the LDV-resistant replicon subpopulation.

### Data Availability

All data generated or analyzed during this study are included in this published article.
